# Sweet and Umami Taste Perception Differs with Habitual Exercise in Males

**DOI:** 10.3390/nu11010155

**Published:** 2019-01-12

**Authors:** Emma L. Feeney, Laura Leacy, Mark O’Kelly, Niamh Leacy, Abbie Phelan, Leah Crowley, Emily Stynes, Aude de Casanove, Katy Horner

**Affiliations:** 1 School of Agriculture and Food Science, Institute of Food and Health, University College Dublin, Belfield, Dublin 4, Ireland; emma.feeney@ucd.ie (E.L.F.); emily.stynes.1@ucdconnect.ie (E.S.); aude.decasanove@ucd.ie (A.d.C.); 2 School of Public Health, Physiotherapy and Sport Science, University College Dublin, Belfield, Dublin 4, Ireland; laura.leacy@ucdconnect.ie (L.L.); mark.o-kelly.1@ucdconnect.ie (M.O.); niamh.leacy@ucdconnect.ie (N.L.); abbie.phelan@ucdconnect.ie (A.P.); leah.crowley@ucdconnect.ie (L.C.)

**Keywords:** taste perception, umami, carbohydrate, sweet, salt, bitter, physical activity, intensity, liking

## Abstract

Taste is influenced by several factors. However, whether habitual exercise level is associated with differences in taste perception has received little investigation. The aim of this study was to determine if habitual exercise is associated with differences in taste perception in men. Active (*n* = 16) and inactive (*n* = 14) males, between ages 18–55, underwent two days of sensory testing, using prototypical taste stimuli of high and low concentrations for sweet, salt, bitter, sour, umami, and carbohydrate (maltodextrin). Mean perceived intensity and hedonic ratings were recorded. Eating behaviour was assessed by the three factor eating questionnaire and food intake by EPIC food frequency questionnaire (FFQ). There were moderate to large differences between the two groups in perceived intensity for sweet taste at the high concentration and umami taste at both high and low concentrations, with active males recording a higher perceived intensity (*p* < 0.05 for all). The active group also recorded a greater dislike for umami low and carbohydrate low concentration (*p* < 0.01). Salt, bitter and sour perception did not significantly differ between the two groups. FFQ analysis showed no difference in % energy from macronutrients between the groups. Eating behaviour traits correlated with sweet taste intensity and umami taste liking, independent of activity status. Results indicated that sweet and umami taste perception differ in active compared to inactive males. Habitual exercise level should be considered in taste perception research and in product development. Whether differences in taste perception could be one factor influencing food intake and thus energy balance with habitual exercise warrants further investigation.

## 1. Introduction

The sense of taste allows us to identify and distinguish between sweet, sour, salty, bitter, and umami qualities [1], perceived on the tongue in the absence of odour. In addition, carbohydrate has recently been described as a taste [2] exemplified by maltodextrin. Taste sensitivity differs between individuals for different taste qualities [3,4]. There is considerable variation in the degree of taste perception, and a wide range of factors, including genetics [3,5], age [6], sleep [7] body mass index [8], anxiety level and neurotransmitters [9], hormonal factors [10], and habitual diet [11], among others, have been associated with differences in taste perception between individuals. Physical activity could potentially influence several of the modifiable factors associated with differences in taste perception. Although the outcomes are variable, some studies have reported alterations in taste perception during and after a single bout of exercise (see References [12,13] for reviews). There is also some limited evidence that habitual exercise may be associated with differences in taste perception [14]. In a study of female swimmers and inactive females, swimmers were found to perceive high-sucrose stimuli as sweeter [14]. However, little other research to date has investigated taste perception and habitual exercise.

Characterising factors influencing food intake in active and inactive individuals is important to gain a greater understanding of the role of physical activity in energy balance [13,15,16]. Sedentary individuals have been proposed to be at a greater risk of overeating due to a lack of physiological regulation of appetite [17] and several aspects of appetite and food intake regulation have been shown to vary depending on habitual physical activity level [18,19,20]. Evidence from both cross-sectional and longitudinal studies suggests physical activity is associated with improved short-term appetite control [19]. Moreover, hedonic responses for high- or low-fat and sweet or savoury foods have been shown to differ between habitual exercisers and inactive individuals [20]. The underlying factors and mechanisms associated with differences in appetite control and food intake with physical activity, however, remain to be fully elucidated.

Understanding whether taste perception differs depending on physical activity level is important, as differences in taste perception could influence food choice or eating behaviour [3,4,21] and may be related to weight status [22,23]. Alterations in taste perception have been linked to weight gain, with a recent longitudinal study demonstrating attenuated sweet and salty taste perception was associated with weight gain in college-aged males [24]. Moreover, taste perception has been proposed as a factor that may influence athletes’ food choices [25]. Determining whether taste perception differs in active and inactive individuals could, therefore, provide greater insight into factors influencing food choice and energy balance. For example, a reduced sensation of sweet or salty taste could potentially render inactive individuals more susceptible to weight gain.

The present study aimed to compare taste perception (taste intensity and liking) between active and inactive males for the five ‘basic’ taste qualities of sweet, sour, salty, bitter, and umami tastes, as well as the more recently proposed carbohydrate taste.

## 2. Materials and Methods

### 2.1. Study Design

Participants in this between-groups cross-sectional design study undertook two separate test mornings one week apart. Ethical approval for the study was provided by the University College Dublin School of Public Health, Physiotherapy and Sports Science undergraduate research ethics committee. The primary outcome measure was taste perception (intensity) and secondary outcomes were liking and identification of taste, anthropometry and body composition, eating behaviour, and habitual dietary intake.

### 2.2. Participants

Thirty men were studied (*n* = 14 inactive and *n* = 16 active) between the ages of 19 and 51 years. Inclusion criteria were: Male, aged 18–55 years, nondiabetic, no medical conditions and not taking medication known to influence taste perception, willing to consume study taste solutions, and nonsmokers. Participants were classified through a screening questionnaire based on their self-reported physical activity patterns over the last 6 months as either inactive (undertaking ≤1 structured exercise session per week and not engaged in strenuous work) or active (undertaking ≥4 structured exercise sessions per week). Individuals who did not fit either category were excluded. One exercise session was defined as at least 40 min of moderate to high intensity activity. Participants were also asked to record the typical intensity, frequency, and duration of each activity per week. These criteria were used as identical to previous studies showing differences in appetite control in active versus inactive individuals [18,26]. We have previously shown these categories to differ in objectively measured physical activity [27]. Sample size calculations were conducted in G*Power [28] using data from a recent study assessing sweet intensity in adults [29], as taste intensity was the primary outcome measure, differences with physical activity in females were previously shown in relation to sweet taste [14], and the most similar literature that provided quantitative data to allow sample size calculation related to sweet taste. To detect a 10-point difference in sweet taste intensity ratings between the two groups in the present study with a power of 80% and a significance level of 5%, 14 individuals were required per group.

### 2.3. Recruitment and Setting

Recruitment was conducted through the distribution of recruitment flyers and emails throughout the university campus. Participants who were eligible to participate based on information provided in the screening questionnaire were invited to participate in the testing sessions. The testing sessions took place in the sensory evaluation suite at UCD’s Institute of Food and Health. Participants were recruited from the 1 January 2018 until the 15 March 2018.

### 2.4. Body Composition Measurement and Taste Perception Assessment Day Protocol

On arrival, all participants provided written informed consent. On both test days, participants attended the laboratory in the morning, having avoided strong-flavoured foods/drinks, such as spicy foods and coffee, for 12 h and strenuous exercise for 24 h, and were instructed to wear light clothing for body composition measurements. Participants’ height measurements were first taken using a stadiometer, followed by weight and body composition using a Tanita body composition analyser (BC-420MA, Tanita Ltd., Yiewsley, UK), which uses bioelectrical impedance (BIA) to assess body composition. Participants were then familiarised with the generalised labelled magnitude scale to assess perceived intensity (gLMS) [30]. The gLMS is a validated scale for assessing taste intensity, according to the standard protocol outlined previously by Green, Schaffer, and Gilmore [31] and Green et al. [32]. A generalised degree of liking scale (gDOL) with labels of ‘neutral’ and ‘strongest liking/disliking of any kind’ was used to assess liking of the stimuli.

#### 2.4.1. Taste Stimuli

Food-grade, prototypical taste stimuli in water were prepared as follows: Sucrose (sweet) (27 mmol/L and 243 mmol/L), citric acid (sour) (1 mmol/L and 9 mmol/L), sodium chloride (salt) (33 mmol/L and 300 mmol/L), quinine (bitter) (0.056 mmol/L and 0.498 mmol/L), monosodium glutamate; MSG (umami) (0.51 g/L and 4.566 g/L) [24], and maltodextrin (carbohydrate) (dextrose equivalent 4.0–7.0, Sigma Aldrich, Arklow, Ireland) (35.5 g/L and 112.4 g/L) [2]. These concentrations were selected to provide ‘low’ and ‘high’ concentrations to allow comparison to recent research [2,24].

#### 2.4.2. Taste Perception Rating

Participants undertook two identical taste sessions spaced one week apart. Previous work has indicated that at least two taste intensity ratings are necessary to achieve reliable estimates of individual taste responsiveness when using the gLMS [33]. Both sessions took place in the mornings, and participants followed identical instructions prior to each visit. The taste stimuli were the same in each session and mean results from the two ratings for each concentration of each taste were the primary outcome used in analyses. Results from the individual test days were also explored to examine the reliability of results at the individual test session. At each session, participants tasted 12 samples (high and low concentrations of the six tastes), served at room temperature in 20 mL medicine cups in a sip-and-spit manner in a randomised block design, presented blinded, using 3-digit randomised codes. The tests were administered on computers located in each sensory booth, using RedJade software (RedJade Software Solutions, LLC, Boulder, CO, USA). Participants were requested to identify the taste from a specified list (‘sweet’, ‘salt’, ‘sour’, ‘bitter’, ‘umami’, ‘carbohydrate’ or ‘unsure’) and then to rate the perceived intensity and then liking of the stimuli on a gLMS and gDOL respectively presented on screen. A 30 s break was enforced with the software between each solution, with a 2-min break after every 4 samples, with water for rinsing provided between each sample.

#### 2.4.3. Eating Behaviour and Habitual Diet

At the end of the taste protocol on the second test day, participants completed a paper-based version of the three factor eating questionnaire (TFEQ) [34] and European Prospective Investigation into Cancer and Nutrition (EPIC) food frequency questionnaire (FFQ) [35].

### 2.5. Statistical Analysis

Statistical analysis was performed using PASW Statistics 24.0 (SPSS Inc., Chicago, IL, USA) and GraphPad Prism version 7.0 for Mac (GraphPad Software, San Diego, CA, USA). To determine if the data of the two groups was normally distributed, the Shapiro–Wilk test was used. For normally distributed data, a parametric independent *t*-test was used; otherwise, the Mann–Whitney U test was used. Pearson’s correlation or Spearman’s rank correlation coefficient were used to determine relationships between variables where appropriate. Effect size (ES: Cohen’s d or r where appropriate) was also assessed. Multiple regression analysis was undertaken to identify the effects of confounding variables such as age and body composition on taste perception. There were no missing data for any outcomes, except for the TFEQ and FFQ for two participants in the active group. Only complete data for the TFEQ was used in analyses (*n* = 14 in both groups). Statistical significance was considered at *p* < 0.05 and data are reported as mean (SD) unless otherwise stated.

## 3. Results

### 3.1. Subject Characteristics

Descriptive data for the active compared to inactive groups are shown in Table 1. Age, height, weight and body mass index (BMI) did not significantly differ between the two groups; however, the active group had a lower body fat percentage.

### 3.2. Taste Identification

Although the study was not designed to assess identification of tastes, we explored whether it differed between the two visits and two groups, as it could potentially influence the results. Overall, taste identification did not differ significantly for any taste and concentration between the first and second session, suggesting no learning effect for taste identification occurred. Mean percentage of tastes correctly identified was greater at the high concentrations than at the lower concentrations for both groups (*p* < 0.05) but did not differ between the two groups (*p* > 0.05). When the individual tastes were compared at the two visits, a greater percentage of the active group correctly identified the umami taste compared to the inactive group (*p* = 0.03). However, there were no significant differences in the identification of all tastes between the two groups for all other tastes and concentrations at the two visits.

### 3.3. Taste Intensity

**Figure 1:**
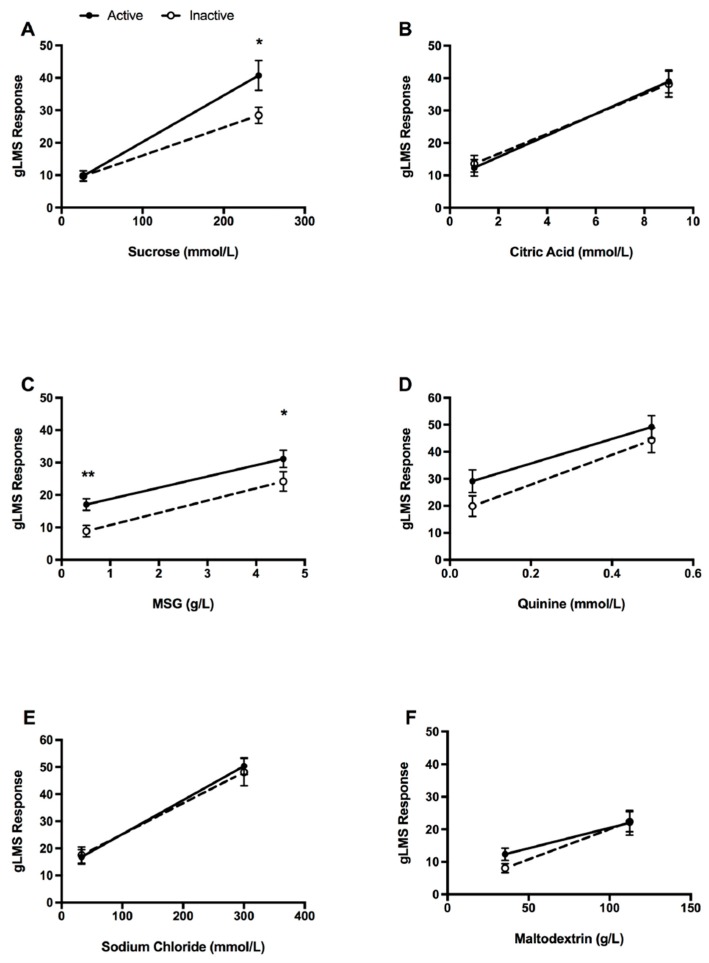
Differences (mean (SE)) in perceived intensity responses on a generalised labelled magnitude scale (gLMS) for high and low concentrations of: (**A**) Sweet, (**B**) sour, (**C**) umami, (**D**) bitter, (**E**) salt, and (**F**) carbohydrate (maltodextrin) taste intensity ratings with physical activity. Solid dark line indicates active group, dashed line indicates inactive group. * *p* < 0.05, ** *p* < 0.01. MSG, monosodium glutamate.

Perceived intensity in the active compared to inactive groups for the six tastes studied are shown in Figure 1. There was a large difference (ES: *d* = 1.63, *p* < 0.05) in perceived intensity for the high-concentration sweet (sucrose) taste between the two groups, with the active group recording a significantly higher intensity rating compared to the inactive group (Figure 1A). Significantly higher intensity ratings were also observed in the active group for the umami (MSG) taste (Figure 1C), with a large difference between the two groups for the low concentration (ES: *d* = 1.18, *p* < 0.01) and moderate difference at the high concentration (ES: *r* = 0.38, *p* < 0.05). Perceived intensity did not significantly differ between the two groups for the low concentration of sucrose, nor for either high or low concentrations of citric acid, quinine, sodium chloride, and maltodextrin (*p* > 0.05 for all; Figure 1). For the latter comparisons, effect sizes were small for all (*d* < 0.30), except for the low concentration of quinine (*d* = 0.60) and low concentration of maltodextrin (*d* = 0.66).

### 3.4. Hedonic Response

Hedonic ratings in the active compared to inactive group for the six tastes studied are shown in Table 2. Apart from the sweet taste, the majority of tastes had negative ratings, indicating varying levels of dislike on the gDOL. There was a large difference between the two groups in liking of the umami low concentration with the active group recording a dislike of the taste, compared to a mean response of a weak liking in the inactive group. Similarly, the active group recorded a dislike for the low-concentration carbohydrate taste, compared to a mean response of a weak liking in the inactive group (Table 2). Although there were no statistically significant differences in hedonic ratings between the two groups for the other solutions, moderate effect sizes were observed for the bitter taste, with a trend towards a greater dislike of the bitter taste in active individuals.

### 3.5. Reproducibility of Taste Intensity Comparisons between Groups at Individual Test Days

Interestingly, carbohydrate perceived intensity was higher at the first visit in the active group (*p* < 0.05) but did not differ at the second visit and therefore was not significantly different when mean ratings were compared. By contrast, umami high concentration intensity ratings did not significantly differ statistically between groups at the individual test days but differed when mean ratings were compared. However, most differences that were observed in mean ratings were also evident at both individual test days, with significant differences or similar trends observed (*p* < 0.1) for high-concentration sweet and low-concentration umami. Moreover, similar to mean ratings, perceived intensity for low-concentration sweet, and both concentrations of sour, bitter, and salty were not different between the two groups at the separate test visits.

### 3.6. Habitual Dietary Intake

There was no difference observed in the percentage of energy from macronutrients, or carbohydrate between the active and inactive group (Table 3), except for fructose, which was higher in the inactive group (*p* = 0.04), and fibre, which trended towards being higher in the inactive group (*p* = 0.05).

### 3.7. Regression Analysis Including Age, BMI, and Body Composition

#### 3.7.1. Taste Intensity

For the sweet taste, physical activity status was the only variable associated with perceived intensity at the high concentration (model adjusted *R*^2^: 0.13; *ß* = −0.39, *p* = 0.03). Age, BMI or percentage of body fat were not independently associated with sweet taste intensity or when included in models for either concentration (*p* > 0.1 for all).

For the umami taste at both concentrations, physical activity status showed the strongest association with perceived intensity. BMI, body fat or age were not associated with differences in umami perceived intensity (*p* > 0.1).

For the sour taste, at the low concentration, both age and body composition were significantly associated with perceived intensity (*p* < 0.01) but not independently in the same model. Moreover, there were no associations between sour taste at the high concentration and age, BMI, percentage of body fat or physical activity status. Bitter and salty tastes also showed no significant associations with these variables at either the high or low concentrations (*p* > 0.1 for all). For the carbohydrate taste, BMI and activity status together in the same model significantly predicted perceived intensity at the low concentration (model adjusted *R*^2^: 0.14; *p* < 0.05; activity status *ß* = −0.30, *p* = 0.099; BMI *ß* = −0.31, *p* = 0.08).

#### 3.7.2. Liking

Sweet and sour taste liking were not associated with physical activity status, age or body composition (*p* > 0.1). However, activity status was associated with liking of the low-concentration umami (*ß* = 0.44, *p* = 0.02), high-concentration bitter (*ß* = 0.37, *p* < 0.05), and low-concentration carbohydrate (*ß* = 0.43, *p* = 0.02) tastes. Liking of the low-concentration salty taste (*ß* = 0.45, *p* = 0.01) and of the high-concentration umami taste (*ß* = 0.45, *p* = 0.01) were both associated with age. Liking of the low-concentration carbohydrate solution was the only variable associated with percentage of body fat (*ß* = 0.45, *p* = 0.01) and, together with activity status in the same model, was associated with 20% of the variance in liking for carbohydrate at the low concentration (model adjusted *R*^2^: 0.20; *p* < 0.05; activity status *ß* = 0.27, *p* = 0.18; body fat *ß* = 0.31, *p* = 0.13).

### 3.8. Regression Analysis with Eating Behaviour

#### 3.8.1. Hunger

Sweet taste perceived intensity for the high concentration was positively associated with the trait hunger (*ß* = 0.39, *p* = 0.04), (i.e., a higher hunger score was associated with greater perceived intensity). This remained significant when included in the same model as activity status. Together, activity status and hunger were associated with 27% of the variance in perceived intensity for sweet taste at the high concentration (model adjusted *R*^2^: 0.27; *p* < 0.01). In addition, hunger was associated with perceived intensity for the high-concentration bitter taste (*ß* = 0.44, *p* = 0.02) and liking for the high-concentration salt taste (*ß* = 0.43, *p* = 0.02).

#### 3.8.2. Disinhibition

Disinhibition was also associated with liking of the high-concentration salt taste (*ß* = 0.49, *p* < 0.01), but not with any other variables.

#### 3.8.3. Restraint

Perceived intensity for the high-concentration bitter taste (*ß* = −0.41, *p* = 0.03), and liking of the low- (*ß* = −0.46, *p* = 0.02) and high- (*ß* = −0.43, *p* = 0.02) concentration umami taste were associated with dietary restraint. As activity status (active or inactive) was also significantly associated with liking for umami low concentration (model adjusted *R*^2^ = 0.19, *ß* = 0.439, *p* < 0.015), they were included in the same model. Together, activity status and restraint accounted for 38% of the variance in umami low concentration liking (model adjusted *R*^2^ 0·38, *p* < 0·001; activity: *ß* = 0·46, *p* < 0·01; restraint: *ß* = −0.47, *p* < 0·001). Dietary restraint was also inversely associated with liking of the high-concentration carbohydrate taste (*ß* = −0.41, *p* = 0.03).

## 4. Discussion

The present findings demonstrate that taste perception intensity differed between active and inactive males. In this cohort, active males reported a greater perceived intensity for both sweet and umami tastes. Given previous evidence of associations between taste perception and food choice [3,4,21], and weight gain [24], these findings may have implications for understanding factors influencing the control of food intake and energy balance with habitual exercise.

Although limited research has investigated associations of habitual exercise with taste perception, our finding of a greater perceived intensity of sweet taste in active males is comparable to a previous study in females. Crystal, Frye, and Kanarek [14] found female swimmers perceived a high-concentration sucrose solution as sweeter compared to inactive females’ using visual analogue scales (VAS). In response to acute exercise, increases, no change, and decreases in acuity of taste and rated preference for tastes have been previously reported, with results appearing to depend on differences in length and intensity of the exercise session and the taste [13]. Regarding sweet taste specifically, Westerterp-Plantenga et al. [36] observed an increase in perceived intensity of taste using VAS for a low-concentration sucrose solution (but not high-concentration) following 2 h of moderate intensity cycling. By contrast, others have reported no change in sweet taste intensity for a sucrose solution, but an increase in intensity of sour taste following 10 minutes of cycling to generate a ‘light sweat’ [37]. However, assessing differences with longer-term interventions and with habitual exercise is also essential, as the repeated effects of regular exercise on physiological and psychological processes of appetite control do not always mimic the acute effects of exercise. Participants in the present study were instructed to avoid strenuous exercise for 24 h before the test sessions to avoid influence of acute exercise on results.

Perceived intensity of umami taste also differed between the active and inactive groups. Perceived intensity of both low and high concentrations of MSG was rated as significantly higher in active males. In a previous study, along with the other ‘basic’ tastes, Horio and Kawamura [38] assessed umami threshold and liking using six different concentration solutions of MSG after moderate-intensity cycling and found no difference compared to pre-exercise in healthy university students. Generally, the effects of both acute and chronic exercise on umami taste perception, however, have not been extensively studied previously.

Several factors could contribute to the differences in sweet and umami taste perception we observed with habitual exercise. Some previous studies have shown habitual diet to be associated with taste perception. For example, sweet taste intensity has been shown to negatively correlate with total energy and carbohydrate and sweet food intake [11], although this was not demonstrated elsewhere [39]. By contrast, higher carbohydrate taste intensity has been positively associated with greater energy and starch intakes, assessed by either FFQ or food diary [2]. In the present study, however, we did not observe differences in the percentage of energy consumed from starch, sugar or other carbohydrate forms, apart from fructose, which was higher in the inactive group, although the limitations of FFQ are recognised.

Eating behaviour traits could also contribute to differences in taste perception between individuals. Dietary restraint and disinhibition have been identified as factors that may influence relationships between adiposity and taste sensitivities to 6-n-propylthiouracil [40]. Therefore, we investigated whether eating behaviours could influence associations between habitual exercise and taste perception. The trait hunger and physical activity were both independently associated with sweet taste intensity perception at the high concentration in the same model. Hunger and restraint were both also associated with perceived intensity of the high-concentration bitter taste (quinine); however, eating behaviours were not related to other perceived intensity ratings. These findings suggest eating behaviour traits may be linked to taste perception of some tastes; however, the traits hunger, restraint or disinhibition do not explain the differences in taste perception observed with habitual exercise.

Although we did not assess hormonal status, hormonal differences could be another mechanism contributing to the differences in taste perception we observed in active compared to inactive males. It is interesting that perception of sweet and umami were the two tastes that differed between the active and inactive groups, indicating the differences in perception were specific mainly to these tastes and not an overall effect on taste function. Both sweet and umami share the same class of taste receptor in the mouth, which initiate a G protein-coupled signalling cascade [10,41,42]. There is now strong evidence that in addition to intestinal signalling, glucagon-like peptide-1 (GLP-1) signalling also occurs within the taste bud [43], and evidence from animal studies indicates GLP-1 signalling has an important role in the modulation of both sweet and umami taste [42]. In the present study, a sip-and-spit technique was used for the tasting of solutions, suggesting any differences in the taste responses observed are influenced by orososensory mechanisms and not a post-oral response to nutrients. Other hormones modified by regular exercise or body composition could also potentially contribute to the differences we observed (see Reference [10] for a review). For example, leptin can inhibit the response to sweet taste [10,44]. An increased circulating leptin (due to a greater body fat percentage) in less active individuals could be one potential mechanism contributing to a reduced intensity of sweet taste. However, BMI or percentage body fat did not moderate the relationship between activity status and sweet taste perception in the present study. Endocannabinoids [45] and glucose levels [46] also influence sweet taste responses and are altered with physical activity [47]. Characterising multiple hormonal factors is warranted in future studies, as it may provide mechanistic insight into differences in taste perception with habitual exercise.

Regarding liking, sweet was the only taste to be positively rated by both groups, adding support that sweet tastes are liked by most individuals [48]. We also observed that in addition to a greater perceived intensity in the active group, active males also had a lower (negative) hedonic rating for the low-concentration umami taste, suggesting the greater perceived intensity could have contributed to a dislike for the taste. In contrast, liking of the sweet and umami high concentration tastes did not differ between the active and inactive groups, despite differences in perceived intensity. Others have also observed no difference in hedonic rating, despite changes in taste perception of simple solutions, including sucrose and quinine sulphate, with acute exercise [36]. One explanation could be the form in which the taste stimuli are provided. For standardisation, all samples here were provided to participants in solutions made up with water. However, when compared to more complex food and beverage matrices, possible differences are likely [49]. The implications of differences in perceived taste intensity with habitual exercise for liking of different foods and food choice warrant further investigation.

Various methodological aspects of the present study should be considered. Participants included males only to eliminate the possible interference of the menstrual cycle on taste perception, and to add to previous research in females [14]. Further research in females is warranted. As body composition has a role in some hedonic aspects of appetite control [15,20], body composition (fat and fat free mass) should also be further considered. Links between taste and body weight or BMI have been previously studied [8,22,23]; however, fewer studies [50] have examined fat or fat free mass. In the present study, BMI did not differ between active and inactive groups, while percent body fat differed significantly. These and other potential modifying variables were included in regression models but did not modify the relationship between physical activity status and taste perception. For logistical reasons, body composition was assessed using BIA. Relationships between taste and body composition have previously been reported using BIA [51]. However, studies have shown variable findings regarding the accuracy of BIA [52,53]. Future studies should explore relationships between taste perception and body composition (fat and free mass) on an individual level using more accurate measures. Measurement of waist circumference would also be relevant. In addition, objective measurement of physical activity should be considered in future studies, to further elucidate relationships with aspects such as energy expenditure and sedentary behaviour. Finally, a significant strength of the study is the use of six tastes, low and high concentrations and two taste sessions, allowing a comprehensive assessment of potential differences in taste perception with habitual exercise. Therefore, the findings can be considered to provide reliable estimates of individual taste responsiveness in active versus inactive males.

In conclusion, these data show sweet and umami taste perception differ in habitual exercisers compared to inactive individuals. There is evidence elsewhere that habitually active individuals have improved energy compensation for energy density of foods [19]. Alterations in taste perception could be one potential mechanism contributing to the regulation of energy balance with exercise. While causal inferences cannot be drawn due to the cross-sectional nature of this study, the findings have implications for researchers and for product development—indicating habitual exercise level should be considered in studies examining taste perception and for consumer selection for product development. Further studies are needed to examine longitudinal responses to exercise intervention and to further explore the underlying mechanisms and implications for food intake.

## Figures and Tables

**Table 1:** Subject characteristics of active (*n* = 16) and inactive (*n* = 14) groups.

	Active	Inactive	*p*-Value	Effect Size (*d*)
*Age* (years)				
Median (IQR)	21 (21.0–22.5)	21 (20.5–25.5)	0.79	*r* = 0.05 ^1^
*Height* (cm)				
Mean (SD)	179.83 (4.91)	181.09 (5.12)	0.50	0.25
*Weight* (kg)				
Mean (SD)	79.03 (8.02)	81.49 (10.96)	0.49	0.26
*BMI* (kg/m^2^)				
Mean (SD)	24.41 (1.93)	24.80 (3.14)	0.68	0.15
*Body Fat* (%)				
Mean (SD)	12.33 (4.30)	18.87 (6.45)	<0.01	1.19

^1^ As data were not normally distributed, *r* is used for effect size.

**Table 2:** Hedonic ratings of tastes assessed using a generalised degree of liking scale (gDOL) ^1^ in active (*n* = 16) compared to inactive (*n* = 14) males.

	Active Mean (SD)	Inactive Mean (SD)	*p*-Value	Effect Size (*d*)
*Sucrose*				
High	38.91 (27.68)	34.75 (28.66)	0.69	0.15
Low	1.44 (10.98)	5.18 (21.85)	0.42	0.22
*MSG* ^2^				
High	−17.06 (31.66)	−16.79 (29.36)	0.98	<0.01
Low	−13.09 (20.31)	2.82 (11.50)	<0.01	0.96
*Citric acid*				
High	−3.72 (34.10)	0.36 (33.31)	0.74	0.12
Low	−5.34 (18.19)	2.07 (22.59)	0.33	0.36
*Quinine*				
High	−57.50 (20.97)	−38.79 (28.06)	0.05	0.76
Low	−34.00 (22.86)	−17.89 (29.02)	0.08	0.62
*Sodium chloride*				
High	−33.16 (43.22)	−29.57 (35.17)	0.50	0.09
Low	−18.72 (16.21)	−11.96 (23.87)	0.73	0.33
*Maltodextrin*				
High	−7.16 (25.5)	−3.32 (22.31)	0.67	0.16
Low	−12.28 (12.76)	0.21 (14.29)	<0.01	0.93

^1^ The labels of the scale were ‘neutral’ and ‘strongest liking/disliking of any kind’. ^2^ MSG, monosodium glutamate.

**Table 3:** Mean energy intake and percentage of energy from macronutrients for active and inactive men (FFQ data).

	Active (*n* = 13) ^1^ Mean (SD)	Inactive (*n* = 13) ^1^ Mean (SD)	*p*-Value (2-Tailed)
Energy Intake kcal/day	2290.7 (841.6)	2018.7 (826.7)	0.41
Macronutrient Intake, % of energy			
Fat	36.4 (3.7)	37.7 (6.6)	0.53
Protein	21.5 (3.6)	19.9 (2.8)	0.22
Carbohydrate	43.8 (4.6)	42.0 (8.8)	0.50
Sugar	18.1 (3.5)	18.7 (4.6)	0.73
Sucrose	6.5 (2.3)	6.7 (1.9)	0.78
Fructose	2.5 (1.0)	3.4 (1.2)	0.04
Galactose	0.1 (0.1)	0.1 (0.1)	0.59
Maltose	0.5 (0.1)	0.5 (0.3)	0.77
Lactose	5.6 (2.0)	4.1 (2.7)	0.11
Starch	25.0 (3.8)	22.5 (6.9)	0.25
Fibre	2.9 (0.8)	3.6 (1.0)	0.05

^1^ FFQ (food frequency questionnaire) data were available for *n* = 26 individuals (*n* = 13 per group). Data were missing for two participants and data for two individuals were removed due to energy misreporting (energy intake >2 SD above or below the mean energy intake were removed as per Low et al. [2].
